# Efficacy of platelet-rich plasma as an adjuvant to surgical carpal ligament release: a prospective, randomized controlled clinical trial

**DOI:** 10.1038/s41598-020-59113-0

**Published:** 2020-02-07

**Authors:** Carmen Trull-Ahuir, Diego Sala, Joaquín Chismol-Abad, Marian Vila-Caballer, Juan Francisco Lisón

**Affiliations:** 1 Department of Nursing, Faculty of Health Sciences, Universidad Cardenal Herrera-CEU, CEU Universities, Valencia, Spain; 2 Department of Orthopedic Surgery and Traumatology, Umivale MATEPSSN.15, Valencia, Spain; 3 Department of Biomedical Sciences, Faculty of Health Sciences, Universidad Cardenal Herrera-CEU, CEU Universities, Valencia, Spain; 4 Department of Medicine, Faculty of Health Sciences, Universidad Cardenal Herrera-CEU, CEU Universities, Valencia, Spain; 5 Centre of Physiopathology of Obesity and Nutrition (CIBERobn), CB06/03 Carlos III Health Institute, Valencia, Spain

**Keywords:** Tendons, Chronic pain

## Abstract

The purpose of this study is to evaluate the efficiency of local platelet-rich plasma (PRP) injection as an adjuvant treatment after carpal ligament release. We conducted a prospective randomized, triple-blinded, controlled trial. Fifty participants with mild to extreme carpal tunnel syndrome (CTS) were randomly assigned either to the PRP (*n* = 25) or the platelet-poor plasma (PPP, *n* = 25) group. After performing open surgical release of the carpal ligament, the inside of the carpal tunnel was irrigated with 3 mL of PRP or PPP according to each participant’s group allocation. The primary outcome was hand grip strength (HGS). Secondary outcomes were the time taken off work after surgery (in days) and scores on the *Wong–Baker Faces Scale*, *Boston Carpal Tunnel Questionnaire*, and *Southampton Wound Assessment Scale*. We evaluated patients before treatment and at 6-weeks. As expected, the pain levels, symptom severity, and functional status improved in all the patients after surgery. However, intragroup analysis revealed that only the participants in the PRP group had regained their pre-operative HGS levels at 6-weeks follow-up. These findings indicate that PRP is an effective adjuvant treatment in patients with mild to severe CTS who require surgery.

## Introduction

Carpal tunnel syndrome (CTS), which affects approximately 3% of the general adult population^1^, is the most commonly diagnosed upper limb peripheral entrapment neuropathy^2^. CTS is characterized by compression of the median nerve (MN) at the wrist as it passes through the carpal tunnel^3^ and typical symptoms include numbness, paresthesias, and pain in areas innervated by the MN. In more severe cases, weakness and atrophy of the thenar muscles (innervated by the MN) can occur which in turn, usually affects the work productivity and quality of life of the affected patient^4–6^.

Depending on the severity of the symptoms, treatments for CTS may be conservative (including the use of medications, a night splint, steroid injections, or physical therapy) or require surgical intervention. Surgical decompression of the carpal tunnel by transection of the transverse carpal ligament is the standard treatment for patients with muscle atrophy, or whose symptoms do not respond to conservative therapies^2,7^. Carpal tunnel decompression is a well-established surgical treatment for CTS whose reported success rate and levels of patient satisfaction are high^8^. Nonetheless, this technique is not exempt from potential complications including: sensitive scar areas, persistent symptoms, neurovascular injury, wound complications, bleeding, pillar pain (pain adjacent to the ligament release site), and reduced grip strength^9,10^. Therefore, adjuvant surgical treatments are worth exploring.

Platelet-rich plasma (PRP) is an autologous blood product comprising concentrated platelets and several growth factors (including transforming growth factor-β, platelet-derived growth factor, epidermal growth factor, vascular endothelial growth factor, insulin-like growth factor-1, fibroblast growth factor, hepatocyte growth factor, nerve growth factor, and keratinocyte growth factor) that play a variety of roles in tissue regeneration and healing. Moreover, PRP is a primary autologous product and so its use avoids concerns about disease transmission or immunogenic reactions. Among its applications, PRP has been used as a safe and novel treatment for clinical peripheral neuropathies^11–18^. Furthermore, local PRP injections have recently been used in the conservative treatment of mild to moderate CTS, with promising success rates^14–20^. However, to the best of our knowledge, no previous studies have investigated the effects of irrigating the carpal tunnel with PRP as an adjuvant to surgical release of the carpal ligament.

Thus, in this study we tested the feasibility of using PRP in the treatment of patients with CTS undergoing surgical treatment. We tested the application of a single dose of PRP immediately after surgery for CTS and compared it to the injection of autologous platelet-poor plasma (PPP). We focused on short-term outcome measures (6 weeks after surgery) which included measuring the amount of leave the patient took from work post-surgery (days), their hand grip strength (HGS), and their *Wong–Baker Faces Scale* (WBFS), *Boston Carpal Tunnel Syndrome Questionnaire* (BCTSQ), and *Southampton Wound Assessment Scale* (SWAS) scores.

## Methods

### Study design

This prospective randomized, triple-blinded, controlled clinical trial (reference number: NCT03548259, 07/06/2018) was designed according to the CONSORT 2010 guidelines^21^ and was conducted at a clinic in Spain from June 2018 to November 2018. The study was approved by the Universidad Cardenal Herrera Human Ethics Committee and followed the ethical guidelines set out in the Declaration of Helsinki. No changes were made to this trial after recruitment of the participants commenced. All the patients enrolled provided written, informed consent for their participation in the study.

Sixty-four patients diagnosed with mild to extreme CTS were assessed for eligibility, and 50 were finally enrolled; they were block-randomized in a 1:1 ratio either into the PRP (*n* = 25) or PPP group (*n* = 25) by an independent researcher using a computerized random number generator. The assignment was concealed from the patients and all the study personnel throughout the duration of the trial.

### Inclusion and exclusion criteria

This study included patients aged 18 to 65 years with clinical and electromyography features compatible with CTS and in whom conservative treatment had not yielded satisfactory results. The exclusion criteria were: recent treatment by local injection of corticosteroids in the carpal tunnel, a concomitant hand pathology, metabolic or endocrine pathology, skin hypersensitivity or dermatological disease, previous surgical intervention for CTS, systemic diseases (vascular, rheumatic, or neoplastic disorders), advanced osteoporosis, pregnancy, hemophilia, or treatment with anticoagulant therapy.

### Carpel tunnel syndrome grades

The severity of CTS was determined according to the neurophysiological CTS classification by Padua *et al*. as: extreme (absence of median motor sensory responses), severe (absence of sensory response and abnormal distal motor latency [DML]), moderate (abnormal sensory nerve conduction velocity [SNCV] and abnormal DML), or mild (abnormal SNCV but normal DML)^22^.

### Procedure

The PRP and PPP fractions were obtained using a GPS III Mini Platelet Concentrate Separation Kit (Biomet Biologics Inc., Warsaw, Indiana, USA) following the manufacturer’s instructions. After providing regional anesthesia, a 27 mL blood sample was drawn from the affected upper limb. Prior centrifugation, a whole blood aliquot was obtained. The blood was then centrifuged to yield 3.5 mL of PRP and 15 mL of PPP; 0.5 mL of each sample were sent to the laboratory for quality-control tests. All the samples were maintained at room temperature in a roller mixer SRT9 (Stuart, Stone, Staffordshire, UK) for not longer than 2 hours prior analysis. The platelet counting was determined using the Coulter Principle with a Hematology Analyzer Coulter ACT 5 Diff AL (Beckman Coulter Inc., Brea, California, USA) following the manufacturer’s instructions.

**Figure 1:**
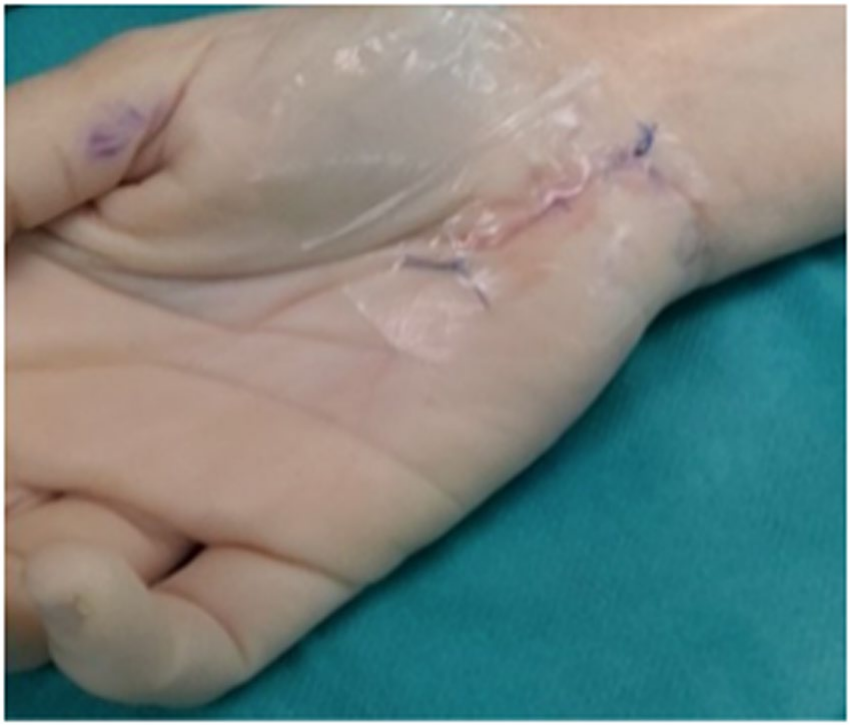
Patient wound after surgical carpel ligament release with a semi-permeable occlusive adhesive dressing which was then covered with a compression bandage.

Open carpal tunnel surgical release of the carpal ligament was performed by experienced surgeons who were blinded to the group allocation of each patient. Once the surgery was completed and the wound sutured, a cannula was inserted through the incision and 3 mL of the PRP or PPP fraction was injected according to the patient’s group allocation. Finally, an occlusive semi-permeable dressing was applied (Fig. 1). All the patients underwent the same standardized post-surgery rehabilitation protocol.

### Outcome measurements

All the outcome measures were recorded in all patients before CTS surgery and 6 weeks after surgery by two trained researchers blinded to the group allocation.

### Primary outcome

The HGS of the affected hand was assessed using a CAMRY hydraulic hand dynamometer (EH101; Camry, Guangdong Province, China). During the measurement, the patients were seated with the shoulder adducted and neutrally rotated, elbow flexed at 90°, and forearm and wrist in a neutral, unsupported position; they were instructed and to squeeze the dynamometer handle as hard as possible for 3 to 5 seconds^23^ and the assessor provided strong verbal encouragement during the tests.

### Secondary outcomes

The secondary outcomes were:

1. User-rated pain levels measured using the WBFS which combines pictures and numbers. Users select the numerical rating assigned to the face that most represents their pain, using a standardized 10-point Likert scale where 0 = no pain and 10 = the worst possible pain^24^. This scale has been shown to have adequate psychometric properties in terms of reliability and validity.
2. The severity of symptoms and the functional status reported by users with CTS, by using the BCTSQ score^25^. Higher scores on this questionnaire represent higher levels of CTS severity and dysfunction. This questionnaire has two distinct scales, one measuring the severity of the symptomology (the BCTSQs) and the other measuring the patient’s functional status (the BCTSQf), each measured on a 5-point Likert scale. Both have been validated in Spanish patients and have adequate psychometric properties^26^.
3. The surgical-wound healing grade using the SWAS score, measured at the 6-weeks follow-up and scored as 0, I, II, III, IV, or V, with higher scores indicating poorer healing^27^.
4. The time the patient took off work (in days) after their CTS surgery.

### Sample size

We conducted a pilot study on 20 participants assigned randomly (at a ratio of 1:1) to the PRP or PPP group. To determine the sample size required to test the significance of the HGS scores between the groups, we performed a power analysis using the G*Power (v3.1.9.2) program. We found that 42 patients would provide 90% statistical power at a 5% significance level (effect size *f* = 0.26). To counteract the expected number of dropouts before completion of the study, we calculated that a total of 50 participants would be required.

### Statistical analysis

We analyzed our data using an intention-to-treat approach. To compare the success of the randomization, we also determined the baseline differences between the groups by using *t*-tests for independent samples, the Mann–Whitney U test for continuous variables, and the χ^2^ test for categorical variables. Two-way mixed analysis of variance (ANOVA) tests were used to compare the effects of the PRP versus the PPP on the outcome measures (HGS, WBFS, BCTSQs, and BCTSQf) between the groups, with time (baseline versus the 6-week follow-up) serving as the within-group factor and the intervention type as the between-group factor. To determine the independent relationship between platelet count and the other outcomes (HGS, WBFS, BCTQs, and BCTQf), we also calculated bivariate correlations for all the patients using the Pearson correlation coefficient.

Assessment of the normality of the data distribution (using the Shapiro–Wilk test) revealed that the leave taken from work after the surgery and SWAS scores at the 6-weeks follow-up were normally and non-normally distributed, respectively. Thus, independent sample *t*-tests (days off from work) and non-parametric Mann–Whitney U tests (SWAS scale scores) were used to compare the study variables between these groups. We performed the statistical analyses using version 19.0 of the SPSS statistical package (IBM Corp., Armonk, NY, USA). Probabilities exceeding 95% (alpha *P*-values less than 0.05) were used as the threshold cut-off for statistical significance.

## Results

**Figure 2:**
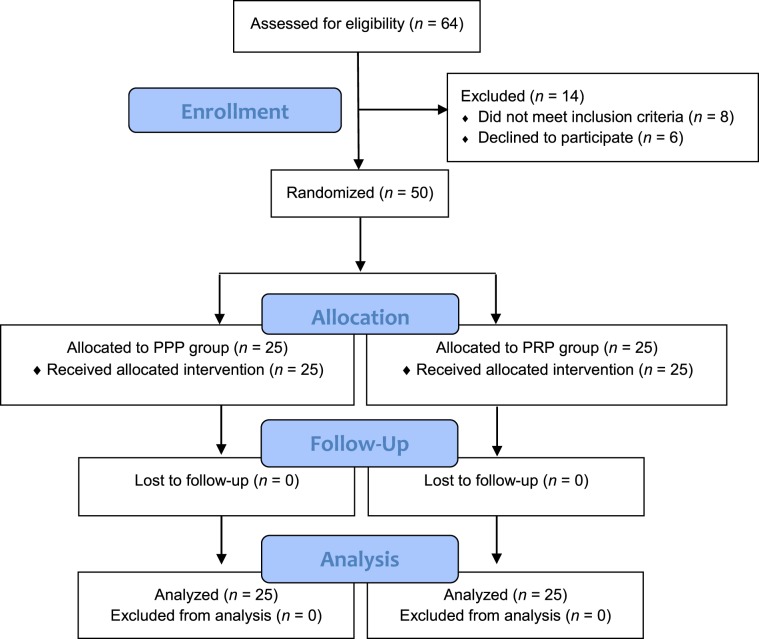
Flow of participants through the trial.

A total of 64 participants were consecutively recruited to this study. Fourteen were not allocated for randomization because they declined to participate (*n* = 6) or did not meet the inclusion criteria because of a concomitant hand pathology (*n* = 4), surgery for a radius fracture (*n* = 1) or for breast cancer on the side affected by CTS (*n* = 1), rheumatic disease (*n* = 1), and insulin-dependent diabetes (*n* = 1). Figure 2 shows the progression of the participants through the trial.

Regarding the blood samples analysis, the mean platelet concentration in the whole blood were 261,000 ± 55,908 platelets/μL in the PRP group and 285,955 ± 74,650 platelets/μL in the PPP group. After centrifugation, the mean platelet concentration were 1,021,488 ± 355,603 platelets/μL in the PRP fraction and 32,439 ± 19,429 platelets/μL in the PPP fraction. The platelet capture efficiency of the device was 50.7% and the factor increase in platelet concentration for PRP was 3.91 ± 1.36. Regarding the doses received by the patients, these were 9.7 × 10^7^ platelets in the PPP group and 3.1 × 10^9^ platelets in the PRP group. All these data agree previous studies^28–31^. No significance correlation was found between the mean platelet count in the PRP with any primary or secondary outcome (HGS: r = 0.080, P = 0.680; WBFS: r = 0.054, P = 0.749; BCTSQs: r = −0.021, P = 0.206; BCTSQf: r = −0.139, P = 0.407). Thus, and according to the *Guidelines for the Use of Platelet Rich Plasma* from the International Cellular Medicine Society and similar studies conducted by Uzun *et al*., higher platelet count does not seem to lead better clinical results^15,32^.

The baseline demographic and clinical characteristics of the study participants are shown in Table 1. At baseline, there were no differences between the groups in terms of age, sex, dominant and affected hand, Padua grading, HGS, WBFS, BCTSQs and BCTSQf, or occupation. As expected, the pain, severity of symptoms, and functional status (WBFS, BCTSQs, and BCTSQf) improved after surgery in both the PRP and PPP group (*P* < 0.001, baseline vs. 6-weeks follow-up; Table 2). In the intra-group analysis after 6-weeks, only the patients in the PPP group showed significant differences in HGS (*P* = 0.016; Table 2). Thus, patients treated with PRP regained their pre-surgery HGS significantly earlier than those in the PPP group, although no differences were found in the between-group analysis at 6-weeks follow-up.

**Table 1:** Baseline demographics and clinical characteristics of the study participants.

Variables	Group	
	PRP	PPP
Age (years)	46.1 ± 6.8	46.4 ± 10.6
Sex (male/female)	6/19	7/18
Dominant hand (right/left)	23/2	23/2
Affected hand (right/left)	13/12	14/11
Padua grading score (mild/moderate/severe/extreme)	(3/9/12/1)	(1/12/11/1)
Occupation		
Restoration and trade-sector workers	10	13
Skilled workers in manufacturing industries	3	3
Installation or fixed machinery operators	2	1
Drivers and mobile machinery operators	2	1
Unskilled services workers	3	3
Agriculture, fishing, and construction workers	5	4
HGS (kg)	20.7 ± 9.9	22.1 ± 9.9
WBFS (0–10)	7.4 ± 2.3	7.6 ± 2.3
BCTSQs (1–5)	3.5 ± 0.8	3.6 ± 0.6
BCTSQf (1–5)	3.4 ± 0.8	3.3 ± 0.9

HGS = Hand Grip Strength; WBFS = *Wong–Baker Faces Scale;* BCTSQ = *Boston Carpal Tunnel Syndrome Questionnaire* (s = severity and f = function).

**Table 2:** Intragroup comparisons.

Variables	Group	Baseline	Follow-up (6 weeks)	Diff. (95% CI)	*P*
HGS (kg)	PRP	20.7 ± 9.9	18.0 ± 10.2	2.7 [−3.1 to 8.5]	0.348
	PPP	22.1 ± 9.9	14.7 ± 6.9	7.4 [1.5 to 13.2]	0.016
WBFS (0–10)	PRP	7.4 ± 2.3	2.6 ± 1.8	4.8 [3.6 to 6.0]	<0.001
	PPP	7.6 ± 2.3	2.5 ± 2.2	5.1 [3.9 to 6.3]	<0.001
BCTSQs (1–5)	PRP	3.5 ± 0.8	1.8 ± 0.6	1.8 [1.4 to 2.1]	<0.001
	PPP	3.6 ± 0.6	2.0 ± 0.6	1.6 [1.2 to 1.9]	<0.001
BCTSQf (1–5)	PRP	3.4 ± 0.8	2.2 ± 0.8	1.2 [0.8 to 1.6]	<0.001
	PPP	3.3 ± 0.9	2.4 ± 0.6	0.9 [0.5 to 1.4]	<0.001

HGS = Hand Grip Strength; WBFS = *Wong–Baker Faces Scale*; BCTSQ = *Boston Carpal Tunnel Syndrome Questionnaire* (s = severity and f = function).

There were no statistically significant differences between groups for the SWAS scores at the 6-weeks follow-up (*P* = 0.609, *Z* = −0.969). Moreover, the median and interquartile range for this variable at 6 weeks was 0 in both cases, indicating that the wound healing was normal in both groups. In addition, both groups also took a similar amount of leave from work after their surgery, although there was a non-statistical significant trend towards a faster return to work by the PRP group compared to the PPP group (110 ± 70 vs. 124 ± 111 days, respectively). Finally, no surgical complications were reported at the 6-weeks follow-up in either the PRP or PPP groups.

## Discussion

This is the first prospective, randomized, triple-blinded controlled trial to explore the efficacy of localized irrigation with PRP as an adjuvant to open carpal tunnel surgery. We demonstrated that participants in the PRP group regained their pre-operative HGS earlier than those in the PPP group. However, contrary to our expectations, we did not find any statistically significant differences between the groups for the remaining variables.

The idea of using PRP as adjuvant to surgical CTS treatment originated in several studies reporting its positive effects on the healing and recovery of structures potentially related to open carpal tunnel surgery. Moreover, PRP also has anti-inflammatory properties in several tissues and pathologies, particularly those related to the skeletal muscle system^33,34^. Thus, we reasoned that patients treated with this adjuvant would also benefit from these effects, particularly in the MN, flexor tendons, and carpal tunnel soft tissues. Our work showed that even though surgery alone would have resolved the ligamental compression present in all our patients, the group injected with PRP appeared to experience a faster reduction in post-surgical inflammation resulting in significantly earlier HGS recovery compared to the PPP group. This improvement may have been related to the enhanced recuperation of the MN, flexor tendons (synovial sheaths), and/or carpal tunnel soft tissues.

### Median nerve

Given that 92% of the patients included in this study reported moderate to extreme CTS, the earlier recovery of HGS among the PRP group may have been associated with neural (i.e. MN) motor improvements. In this sense, several growth factors have been suggested to play a positive role in the regeneration of injured peripheral nerves and the elimination of neuropathic pain^35–41^. Indeed, based on data from animal models, some researchers have hypothesized that PRP specifically promotes angiogenesis, neurogenesis, and regeneration by directly acting upon the nerve itself^42–46^.

PRP also has an anti-inflammatory effect in nervous tissue and can attenuate inflammation in mouse models of neurodegenerative diseases^47,48^. Moreover, it can reduce MN swelling and reduce the subsynovial connective tissue thickness within the carpal tunnel in a rabbit model of MN injury^45^. Additionally, there is mounting evidence that PRP positively affects human peripheral nerve regeneration^11–18^, and it has recently been used in the conservative treatment of mild to moderate CTS^14–20^. For instance, in a quasi-experimental uncontrolled study, an ultrasound-guided injection of 1–2 mL of PRP in 14 patients with mild CTS produced positive outcomes in terms of pain at 1 and 3 months’ follow up^14^. Compared to a steroid control, PRP injection in a non-randomized, single-blinded trial involving 40 patients with minimal to mild CTS resulted in significantly improved symptomology (BCTSQs) and functional status (BCTSQf) at 3 months but not at the 6 month follow-up^15^.

Another well-designed prospective randomized, single-blinded, controlled trial by Wu *et al*. tested the effects of ultrasound-guided injection of 3 mL of PRP in 60 patients with mild to moderate CTS^16^. At 6 months post-treatment, these authors showed significant reductions in pain (measured with the visual analogue scale), global BCTSQ scores, and the cross-sectional area of the MN compared to the night splint control group; they concluded that PRP is safe and effectively relieves pain and improves disability in these patients. Similarly, the ultrasound-guided injection of 2 mL of PRP in 50 patients with mild to moderate CTS improved disability of the shoulder, arm, and hand (Q-DASH questionnaire) at 4 and 12 weeks’ follow-up, in a prospective, randomized, placebo-controlled trial^17^. More recently, Güven *et al*. found that a single injection of 1 mL of PRP combined with the use of a night-splint for 4 weeks improved the electrophysiological metrics in these CTS patients and reduced the cross-sectional area of the MN compared with the night-splint only control^18^. Finally, in patients with CTS, a single injection of 2 mL of PRP in 18 patients compared to the injection of corticosteroids in 18 controls resulted in significant improvements in the VAS and BCTSQ scores at the 1 and 3-month follow-up, but produced minimal differences in the motor and sensory nerve conduction studies^19^.

Despite these aforementioned studies, only the thenar muscles were affected by our treatment while several other muscles also contribute to HGS. This makes it unlikely that enhanced MN recovery was responsible for all of the HGS improvement we saw in this study. Moreover, even though the use of PRP as a conservative treatment for mild to moderate CTS is promising, these results should be taken with caution because there is still no consensus about its effects. Some studies have presented limitations or suggest that different doses may lead to different outcomes and univocal guidelines about the preparation PRP are not yet available. In addition, the molecular mechanism of PRP in this context has not yet been fully elucidated.

### Carpal tunnel flexor tendons

It is also possible that the earlier recovery of HGS among the patients injected with PRP in our study was related to improved flexor tendon healing and reduced inflammation in this group. In occupational settings, CTS is usually caused by repetitive movements of the wrist and fibrous hypertrophy of the synovial flexor sheath^49^; this flexor tenosynovitis often results in intracarpal swelling and MN compression, eventually resulting in or perpetuating CTS^50^. All patients in our study were manual occupational workers recruited from a mutual insurance company who fit this previously mentioned profile. Injection of PRP in these patients may have reduced the carpal tunnel tendon and MN inflammation present in this cohort, thus benefitting, not only the muscles innervated by the distal branches of the MN, but all the irrigated flexor tendons within the carpal tunnel which are the primarily responsible for transferring tensile loads to hand grip (digitorum profundus and superficialis tendons). In this sense, several *in vitro* and *in vivo* studies have shown that PRP has anti-inflammatory effects and improves tendon healing, and have elucidated its molecular mechanism of action in this context^51–61^. Nonetheless, other human studies reported that PRP had no beneficial effects on tendon healing^62–66^, or only found them after longer follow-ups^67–70^. Thus, the controversy surrounding the clinical efficacy of PRP leaves the tendon-healing explanation for the earlier recovery of HGS in our study partially in question.

### Carpal tunnel soft tissues

Finally, the positive effects of PRP could also be associated with improved carpal tunnel soft tissue wound healing. There is a growing consensus for the positive effects of PRP as an adjuvant therapy to aid the healing of surgical wounds and injuries. In fact, the growth factors present in PRP are involved in every stage of wound healing^71–73^, and several reviews^74–78^, systematic reviews^79–81^, and clinical studies^82–84^ have reported their involvement in enhanced soft-tissue healing outcomes. Our results did not show statistically significant differences in the SWAS scores between the groups because they both obtained the best possible score. Moreover, the SWAS evaluates the external appearance of healing wounds but may not accurately assess the healing of the deeper tissues in contact with PRP. Furthermore, we evaluated the healing at 6-weeks and so we have no data about the effectiveness of PRP in the initial stages of healing.

As expected, and in agreement with the effects reported elsewhere^6,85,86^, surgical release of the carpal ligament nearly normalized the pain (WBFS) and symptomology (e.g. numbness or tingling sensations; BCTSQs) and low or medium hand-strength motor function of activities (e.g. writing, buttoning up clothes, holding a book while reading, gripping of a telephone, opening jars, performing household chores, carrying a grocery basket, or bathing and dressing; BCTSQf) scores^87,88^ in both groups. However, in agreement with the results reported by Malahias *et al*.^17^, Wu *et al*.^16^, and Güven *et al*.^18^ these improvements did not differ between these two patient groups in our study.

The use of dynamometry is a well-documented method for objectively quantifying motor outcomes and HGS and is a particularly useful for evaluating the outcome of carpal tunnel release surgery^89^. Unlike the WBFS and BCTSQs, the HGS test measures the motor component of all the muscles involved in forming a hand grip. In addition, unlike the BCTSQf, the HGS test measures maximum-effort motor function by verbally encouraging patients to squeeze the dynamometer as hard as possible for 3 to 5 seconds. Therefore, even though there were no significant differences between the groups in our study when measuring resting pain (WBFS), symptomology (e.g. numbness or tingling sensations; BCTSQs) and low or medium hand-strength motor function of activities (BCTSQf), it is plausible to speculate that the PRP group felt comparatively less discomfort during the HGS test than the PPP group.

This may be because of better healing of the soft tissues (skin, subcutaneous tissue, palmar fascias, and transverse carpal ligament) affected during carpal tunnel surgery, allowing them to recover their pre-surgery HGS levels earlier. Reduced post-surgical HGS may also result from muscle contraction-related pain, either directly or indirectly via contraction of the muscles originating from the flexor retinaculum; these would transmit tension to the cut and healing transverse carpal ligament, causing increased pillar region and/or scar sensitivity^90^. In the same vein, Ludlow *et al*.^91^ pointed out that grip and post-operative scar tenderness predict the return to manual work. In our study the PRP group returned to work an average two weeks earlier than the PPP group, although this difference was not statistically significant. In short, patients treated with PRP after open carpel tunnel surgery may benefit both from enhanced healing and diminished inflammation, resulting in reduced discomfort at maximum effort levels when using a dynamometer to test HGS.

It is also important to mention the limitations of this study. First, the follow-up periods should be reconsidered in order to properly evaluate both the short-term effects of PRP in inflammation and healing, and its long-term efficacy in reducing the severity of symptoms and increasing functional status. Moreover, the tissue fibrosis should be evaluated to rule out unwanted side effects due this therapy. Second, the leukocyte and erythrocyte counting were not included in our study. Others have published the cellular content of PRP after blood centrifugation with the GPS III Mini Platelet Concentrate Separation Kit, reporting a leukocyte and erythrocyte content ranging 23.7–34.4 × 10^3^ and 1–4.82 × 10^6^ respectively^28–31^. Considering, on the one hand the platelet data, and on the other hand the reliability of the device, it could be presumed similar results in our samples. Nevertheless, further experiments are required to analyze the content and influence of all the cellular components of PRP in the surgical treatment of CTS. Third, we did not evaluate the mechanism of PRP action in this study because it was beyond the scope of our design and therefore this aspect should be explored in future studies. Finally, future work should aim to standardize PRP preparation methods and optimize the treatment procedures using it.

In conclusion, this study shows the feasibility of using PRP to treat patients with CTS scheduled for treatment by open carpal ligament release surgery. Because the procedure involves injecting PRP immediately after suturing the wound, it could easily be adapted for use in endoscopic carpal tunnel surgery. Our results showed that patients in which the carpal tunnel was irrigated with PRP as an adjuvant to surgical release of the carpal ligament regained their pre-operative HGS earlier than patients who received the placebo control. However, PRP did not improve pain or the severity of symptoms or functional status more effectively than the control at the 6-week follow-up. Exploration of the efficacy of PRP as an adjuvant to surgical CTS treatment merits further study, although future experiments should address the mechanism by which PRP irrigation aids the early recovery of HGS and should optimize the conditions of its use to maximize the benefits of this potential therapy.

## Data Availability

Data available on request from the authors.
